# Impact of chronic khat chewing on liver function tests and fasting blood glucose levels among adult male khat chewers

**DOI:** 10.1038/s41598-026-54220-w

**Published:** 2026-05-28

**Authors:** Abera Abreham, Abush Getaneh, Gobena Dedefo, Mekdes Alem, Andualem Bayih, Melaku Tsegaye, Birku Gashaw, Geleta Gemechu, Yazal Abay, Abebe Edao Negesso, Mistire wolde, Samuel Kinde

**Affiliations:** 1 Department of Medical Laboratory Science, College of Health Science, Dilla University, Dilla, Ethiopia; 2 Department of Medical Laboratory Science, College of Medicine and Health Science, Addis Ababa University, Addis Ababa, Ethiopia; 3 School of Medical Laboratory Sciences, College of Health Sciences and Medicine, Wolaita Sodo University, Wolaita Sodo, Ethiopia; 4 Department of Medical Laboratory Science, College of Medicine and Health Science, Wolkitie University, Wolkitie, Ethiopia; 5 Department of Medical Laboratory Sciences, College of Medicine and Health Sciences, Rehoboth Harme College, Asella, Ethiopia

**Keywords:** Khat, Liver function, Fasting blood glucose, Dilla town, Ethiopia, Biochemistry, Diseases, Health care, Medical research

## Abstract

Khat (*Catha edulis*) has been widely chewed in the Middle East and Eastern Africa, particularly in Ethiopia. However, its potential adverse effects are not investigated in well-controlled, community-based studies. Therefore, this study assessed the impact of khat chewing on blood glucose and liver function tests. A community-based comparative cross-sectional study was conducted among 100 adult male chronic khat chewers and 100 non-khat chewers in Dilla Town, Southern Ethiopia, from June to September 2023. Serum fasting blood glucose and liver function parameters, including aminotransferases, alkaline phosphatase, total protein and total and direct bilirubin, were analysed using an automated clinical chemistry analyser (EXL 200 Siemens, Germany). Fisher’s exact test, Mann–Whitney U test, Kruskal–Wallis test, and spearman correlation analyses were performed as appropriate. A *p*-value < 0.05 was considered statistically significant. Khat chewers had higher aminotransferase enzyme levels (aspartate aminotransferase and alanine aminotransferase) and fasting blood glucose than non-khat chewers (*p* = 0.007, *p* =  < 0.001, and *p* = 0.002, respectively). Khat chewers’ alanine aminotransferase levels were positively correlated with chewing frequency (days/week), duration (in years), and dose, with Spearman’s rho (*p*) values of 0.488 (< 0.001), 0.679 (< 0.001), and 0.323 (< 0.001) respectively, and duration of khat chewing had a positive correlation with fasting blood glucose with Spearman’s rho (*p*) values of 0.282 (0.004). The present findings indicated that aminotransferase enzymes and fasting blood glucose levels were significantly higher among khat chewers than non khat chewers. Dose, duration, and frequency of khat chewing also had a positive correlation with aminotransferases and fasting blood glucose levels.

## Introduction

Kha*t (Catha edulis)* is a herbal drug cultivated as a green bush, and it is mostly grown in Southern Arabia and East Africa. Ethiopia is the prominent khat-producing country in the world, and khat chewing is a widespread practice among adults in the country^1^. Khat consists of a large number of active ingredients such as cathinone, cathine, and norephedrine among which cathinone is the principal and predominant psychoactive ingredient found in khat’s fresh leaves and twigs, as confirmed by phytochemical analysis^2^.

The World Health Organization (WHO) expert committee on drug dependence categorized khat as a drug of abuse in 2006, and those who abuse it may experience a variety of health issues^3^.

The liver is a major organ responsible for metabolizing, detoxifying, and eliminating substances from the body. The status of the liver is assessed and evaluated by liver function tests, such as liver enzymes, total protein, direct and total bilirubin^4^.

Regular and regular khat chewing increases exposure to reactive oxygen species, which in turn damage cellular membranes. This oxidative stress compromises membrane integrity, leading to increased permeability and leakage of intracellular components into the bloodstream^5^. Scientific case reports indicated that chronic khat chewing induced chronic liver injuries, inevitable progression to fibrosis, and even cirrhosis. The following were indications that khat chewing caused liver injury in the cases. Firstly, cessation of khat chewing led to recovery, and re-exposure to khat caused further liver damage. Secondly, a high concentration of cathinone was detected in the serum of khat chewers, and no other cause for their liver condition was revealed^6–8^. The study conducted by Maria Joao Valente et al. 2016 showed hepatic damage caused by cathinone, involving mitochondrial malfunction and oxidative stress generation^9^. A case–control study from Ethiopia in 2018 indicated the existence of a strong association and a causal relationship between khat chewing and the development of liver diseases. According to the study, khat chewers had a 2.64-fold greater risk of developing liver diseases than non-khat chewers^10^.

There are limited studies and contradictory findings about the effect of khat on blood glucose. Some studies suggested that khat has a hyperglycemic effect, while other studies stated the hypoglycemic effect of khat. Some studies reported that khat has no significant impact on blood glucose levels. There is no conclusive evidence about the effect of khat chewing on fasting blood glucose levels^11^.

Cathinone has an adrenergic action, raises plasma catecholamine levels, and inhibits insulin’s effects. As a result, the capacity of the pancreatic beta cells to make insulin is repressed, glucagon is released, glycogenolysis in the liver is triggered, adrenocorticotropic hormone (ACTH) is released, and blood sugar levels are frequently raised^12^. A study conducted in the Jazan region of Saudi Arabia indicated that khat chewing is significantly associated with diabetes mellitus development. The research found that khat chewers had more than a threefold greater risk of developing diabetes than non-khat chewers^13^. A study from Yemen also showed that khat is a risk factor for the development of diabetes mellitus^14^. The WHO and Ethiopian Public Health Institute report on non-communicable disease risk factors and the prevalence of specific non-communicable diseases demonstrated that khat chewing is a risk factor for hyperglycemia^15^. This study aims to determine the impact of khat (*Catha edulis*) chewing on fasting blood glucose and liver function by comparing fasting blood glucose and liver function tests between healthy adult male chewers and non-khat chewers in Dilla town, Southern Ethiopia. Additionally, the research seeks to identify specific factors associated with variations in these biochemical markers among the khat chewers during the period from June to September 2023.To the best of the researcher’s knowledge, there is no published literature examining the effect of khat on blood glucose levels and liver function tests among khat chewers in our study area, Dilla town. Therefore, the present study provides evidence about liver function tests and blood glucose levels and khat chewing correlation. Furthermore, the findings of the present study will assist the clinician in the management of liver and blood glucose in adult male khat chewers. The potential health problems of khat are less emphasized in Ethiopia and this study will give more awareness on the health impacts of khat on the liver and blood glucose. Moreover, the associated factors found in the current study will have an advantage in how khat chewing education is designed, targeted, and implemented to prevent health issues related to khat chewing. Generally, in the scarce data situation on khat’s health impacts in Ethiopia, this study provides helpful information about the impacts of khat chewing on liver function and blood glucose levels.

## Materials and methods

### Study area, design and period

The present study was a community-based comparative cross-sectional study conducted in Dilla town from June to September 2023. Dilla town is located in the southern part of Ethiopia at an elevation of 1570 m above sea level and is found about 360 km from Addis Ababa, the capital of Ethiopia.

### Study population

The present study involved apparently healthy adult male khat chewers and non-khat chewers who had been residing in Dilla town for more than 6 months and who volunteered to give a fasting blood sample. Khat chewers were recruited from local khat sellers using a purposive sampling technique, and non-khat chewers were recruited from university hospital staff and residents willing to participate in this study.

### Inclusion criteria

The study included males aged between 18 and 60 who willingly agreed to participate in study. 

### Exclusion criteria

Individuals with hepatitis, known liver diseases, diabetes mellitus, or metabolic disorders were excluded. In addition, those taking medication or who did not attend data collection in a fasted state were excluded.

### Study variables

*Dependent variables*: The levels of liver function tests (Aspartate Aminotransferase (AST), Alanine Aminotransferase (ALT), Total Protein (TP), Direct Bilirubin (DBI) ,and Total Bilirubin (TBI) and fasting blood glucose (FBG).

*Independent variables*: Socio-demographic characteristics (age, sex, marital status, and educational status); behavioural factors (alcohol and smoking): physical activity intensity; and khat chewing characteristics (duration, frequency and bundle of khat).

### Sample size determination and sampling technique

The sample size was calculated using a formula for comparing two means based on previous data from Yemen. After adjusting for a 10% non-response rate and deciding to maximize statistical power, two hundred study participants, consisting of one hundred adult male khat chewers and one hundred adult male non-khat chewers, participated in this study. A convenient sampling method was applied to select the study participants.

### Demographic and clinical data collection

The study participants’ sociodemographic data were collected with a structured, pretested, and translated questionnaire. The study participants were directly interviewed by data collectors to complete the questionnaire.

### Blood specimen collection

After fasting for 8–12 h, about 5 ml of blood sample was collected by an experienced laboratory technologist with a serum separator tube from each participant through an aseptic/sterile technique. The serum was obtained from the collected blood sample by centrifuging at 3000 rpm for 5 min after 30 min of blood sample collection. The serum levels of liver function tests and fasting blood glucose were analyzed using Siemens EXL200 chemistry analyzers at Dilla University general Hospital.

### Data quality assurance

The principal investigator was under close contact and supervision throughout the data collection process. Standard pre-analytical, analytical, and post-analytical protocols were consistently maintained to ensure data quality and reliability. To minimise potential bias, the study employed strict eligibility criteria, excluding individuals with chronic illnesses or those on medication to isolate the effects of khat chewing from confounding health factors, and interviews were conducted in private settings using standardised questionnaires.

### Data analysis and interpretation

The data were entered into EpiData version 4.6, then exported to and analyzed using SPSS version 27. The frequency of both khat chewers’ and non-khat chewers’ data were analyzed using descriptive statistics. Fisher’s exact test was used to assess the association between the categorical variables of khat chewers and non-khat chewers. A Mann–Whitney U test was used to compare liver function test results and fasting blood glucose levels between khat chewers and non-khat chewers. The correlation between the level of liver function tests and fasting blood glucose level with continuous variables was analyzed using Spearman’s correlation. To compare liver function tests and fasting blood glucose levels across categories of khat-chewing duration, the Kruskal–Wallis test was carried out using R version 4.5.2 software. The results of the analysis were presented with tables and figures, where appropriate. A p-value of less than 0.05 was considered statistically significant.

## Results

### Socio-demographic and behavioural characteristics study participants

This study involved two hundred (200) apparently healthy adult male study participants (100 khat chewers and 100 non-khat chewers). The median age of khat chewers was 36 years (IQR = 12), whereas the median age of non-khat chewers was 34 years (IQR = 14). There was no statistically significant difference in age distribution between khat chewers and non khat chewers (*p* = 0.169) (Table 1).

**Table 1:** Socio-demographic and behavioural characteristics of study participants in Dilla town, Southern Ethiopia, 2024 (N = 200).

Variables	Categories	KC (n = 100)	NKC (n = 100)	Total (%)	P-value*
Age	18–30 31–43 *\ge* 44	22 (22%) 56 (56%) 22 (22%)	35(35%) 46 (46%) 19 (19%)	57 (28.5) 102 (51) 41(20.5)	0.13
Educational status	No formal ed Primary School *\ge* Secondary	11 (11%) 68 (68) 21 (21%)	12 (12%) 59 (59%) 29 (29%)	23 (11.5) 127 (63.5) 50 (25)	0.38
Marital status	Single Married divorced	25 (25%) 70(70%) 5 (5%)	22 (22%) 75 (75%) 3 (3%)	47 (23.5) 145 (72.5) 8 (4)	0.64
Smoking status	Yes No	18 (18%) 82 (82%)	11 (11%) 89 (89%)	29 (14.5) 171 (85.5)	0.23
Alcohol consumption	Yes No	37 (37%) 63 (63%)	29 (29%) 71 (71%)	66 (33) 134 (67)	0.29
Physical Activity	Vigorous Moderate Less active Sedentary	13 (13%) 12 (12%) 12 (12%) 63 (63%)	12 (12%) 18 (18%) 23 (23%) 47 (47%)	25 (12.5) 30 (15) 35 (17.5) 110 (55)	0. 07
Dairy products and fat consumption	< 3times / week ≥ 3times / week	72 (72%) 28 (38%)	62 (62%) 38 (38%)	134 (67) 66 (33)	0.18
Fruit/vegetable consumption	Low Sufficient	61 (61%) 39 (39%)	60(60%) 40(40%)	121 (60.5) 79 (39.5)	0.89

Abbreviations: KC:—khat chewers, NKC: -Non-khat chewers, *: -fisher’s exact test.

### Khat Chewing characteristics among Khat Chewers

Forty-four (44%) of khat chewers chewed for 8–14 years, while Forty-eight (48%) chewed daily. (Table 3) In addition, 70(70) of the khat chewers chewed less than one bundle per day (Table 3).

## Comparison of liver function tests and fasting blood glucose levels among khat chewers and non-khat chewers

The levels of aminotransferase enzymes (AST and ALT) and fasting blood glucose (FBG) were statistically significantly higher among khat chewers compared to non-khat chewers. The levels of ALP, TP, DBI, and TBI were not statistically significantly varied between the two groups (Table2).

**Table 2:** Khat chewing status among khat chewers in Dilla, Southern, Ethiopia, 2024 N (100).

Characteristics	Category	N (%)
Duration (years)	*łe* 7 yrs	24 (24%)
	8-14yrs	44 (44%)
	*\ge* 15yrs	32(32%)
Frequency (days per week)	*łe* 6 days per week	52(52%)
	7 days	48(48%)
Bundle per day	< 1 bundle per day	70(70%)
	*\ge* 1 bundle per day	30(30%)
Chewing style	Water	59(59%)
	Soft drinks	13(13%)
	Peanut	17(17%)
	Others (milk, coffee, tea)	11(11%)

### Correlations of liver function tests and fasting blood glucose level with khat-chewing characteristics (N = 200)

The duration of khat chewing showed a strong positive correlation with ALT and a weak association with AST and fasting blood glucose (FBG). The frequency of khat chewing demonstrated a moderate positive correlation with ALT and a weak correlation with FBG. The quantity of khat consumed (measured as bundles) was very weakly and positively associated with ALT (Table 3).

**Table 3:** Comparison of liver function tests and fasting blood glucose among khat chewers and non-khat chewers in Dilla town, Southern Ethiopia, 2024 (N = 200).

Parameters	Khat chewers (KC)	Non-khat chewers(NKC)	P-value
	median ± IQR	Median ± IQR	
Aspartate aminotransferase (IU/L)	30.50 ± 10	27 ± 10	**0.007**
Alanine aminotransferase (IU/L)	30 ± 16	25 ± 10	** < 0.001**
Alkaline phosphatase (IU/L)	81.5 ± 33	77 ± 24	0.098
Total protein (g/dl),	7 ± 0.9	7.2 ± 0.9	0.113
Direct bilirubin (mg/dl),	0.1 ± 0.1	0.1 ± 0.1	0.139
Total bilirubin (mg/dl),	0.5 ± 0.3	0.5 ± 0.3	0.095
Fasting blood Glucose (mg/dL)	83 ± 11	79.5 ± 10	**0.002**

IQR: Interquartile range, Mann–Whitney test was used for comparison.

### Effect of khat chewing duration on AST, ALT and FBG

**Fig. 1:**
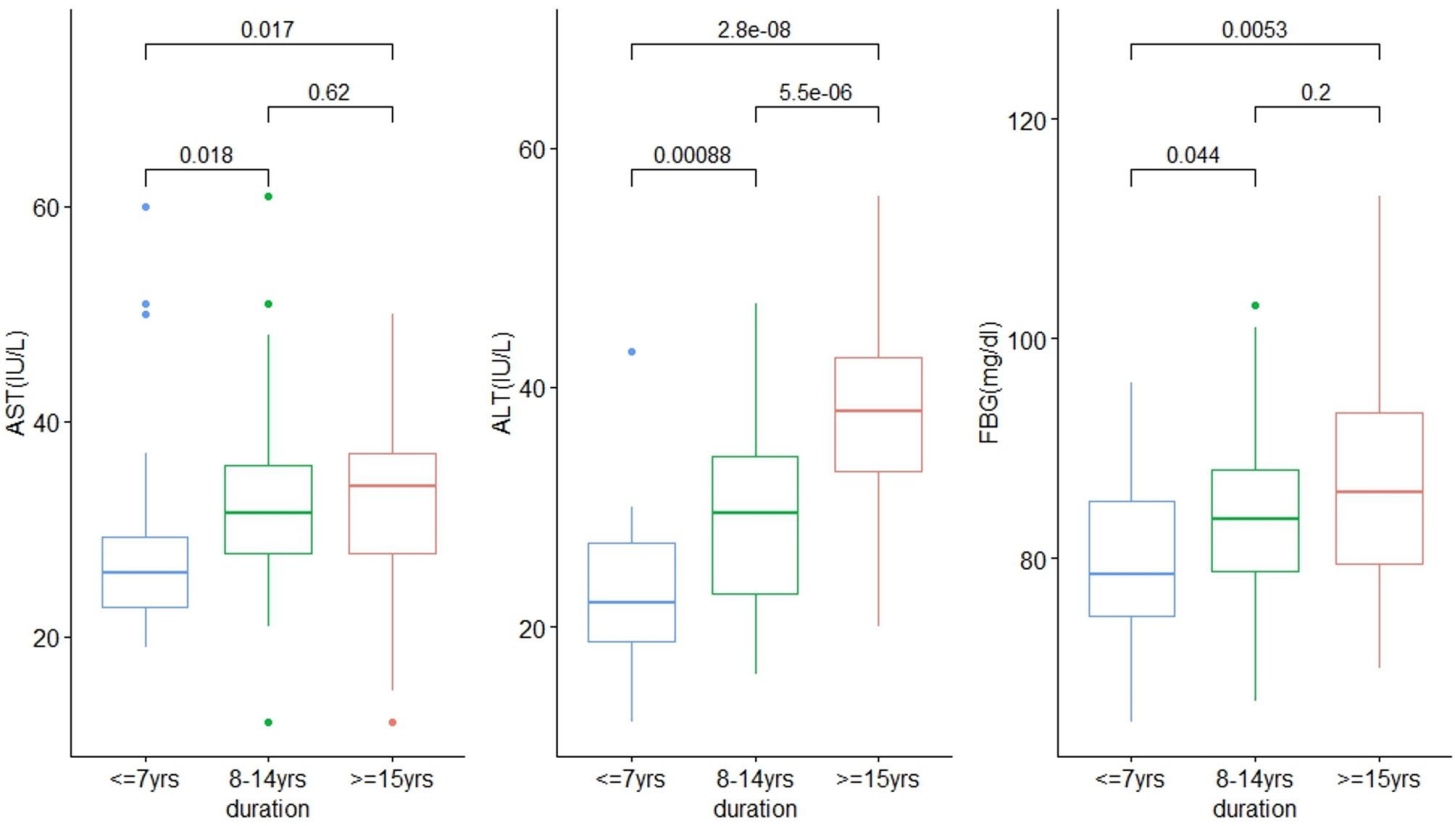
Effect of khat chewing duration on AST, ALT and FBG. *Note*: Kruskal–Wallis test was used to compare AST, ALT and FBG in group of duration and analysed with R version 4.4.0 software.

The duration of khat chewing had a positive relationship with transaminase enzymes (AST and ALT) and fasting blood glucose levels. As the duration of khat chewing increased, the levels of transaminase enzymes and FBG also increased (Fig. 1).

### Effect of khat chewing duration on ALP, TP, DBI And TBI

**Fig. 2:**
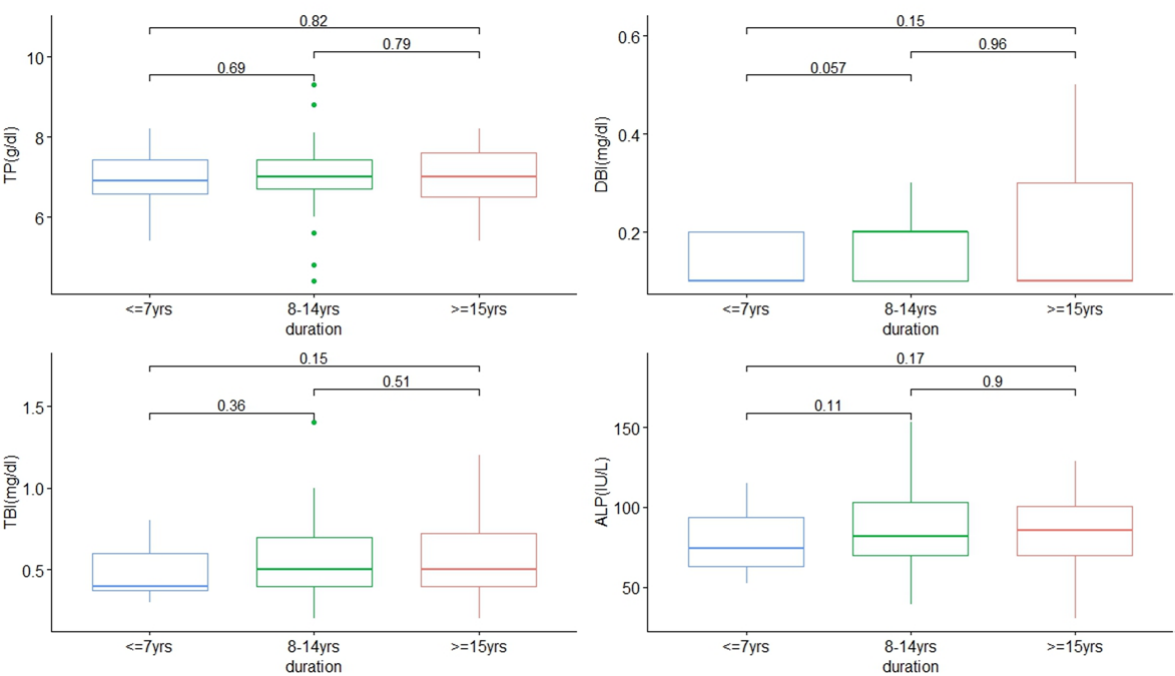
Effect of khat chewing duration on the levels of ALP, TP, DBI and TBI. *Note*: Kruskal–Wallis test was used to compare ALP, TP, DBI and TBI in group of duration and analysed with R version 4.4.0 software.

There was no statistically significant difference in ALP, TP, DBI and TBI levels among each duration of khat chewing (Fig. 2).

## Discussion

Khat contains primarily cathinone as a main psychoactive component; However, it is not the only bioactive compound. Additional alkaloids such as cathine and norephedrine, along with flavonoids, terpenoids, tannins, and phenolic compounds, may also contribute to its physiological effects, including potential alterations in blood glucose regulation and liver enzyme activity^16,17^.

The statistically significant elevations in transaminase enzymes (AST and ALT) observed among khat chewers compared to non-chewers in the present study are consistent with findings reported from the Jazan region of Saudi Arabia^18,19^ and Yemen^20^. The increased level of transaminase enzymes (AST and ALT) among khat chewers could be due to the components in khat that may have caused the production of reactive oxygen species in khat chewers, which may lead to the disruption of hepatocytes’ cytosolic and mitochondrial membrane integrity due to accumulated free radicals generated during the metabolic activities of khat^21,22^. (Table 4).

**Table 4:** Spearman Correlation (rho) of liver function tests and fasting blood glucose level with Khat-Chewing Characteristics (N = 200).

Variables	AST (IU/L) rho (p)	ALT (IU/L) rho (p)	ALP (IU/L) rho (p)	TP (g/dl) rho (p)	DBI (mg/dl rho (p))	TBI (mg/dl) rho (p)	FBG (mg/dl) rho (p)
Duration	0.249 (0.012)*	0.679 (< 0.001)***	0.089 (0.377)	− 0.11 (0.912)	0.137 (0.173)	0.111 (0.273)	0.282 (0.004)**
Frequency	0.156 (0.121)	0.488 (< 0.001)***	0.044 (0.664)	− 0.109 (0.281)	0.083 (0.413)	0.077 (0.448)	0.242 (0.015)*
Bundle	0.116 (0.250)	0.323 (< 0.001)***	− 0.083 (0.411)	− 0.024 (0.811)	0.118 (0.241)	0.133 (0.187)	0.102 (0.344)

rho:—spearman correlation coefficients, *P*-values: *** (*p* < 0.001), ** (*p* < 0.01), * (*p* < 0.05).

In contrast to the statistically significant increase of the transaminase enzymes (AST and ALT) in the current study, the study conducted in Kenya^23^ and Yemen^24^ showed a statistically non-significant difference in transaminase enzymes (AST and ALT) among khat chewers compared to non-khat chewers. However, the study conducted in Kenya indicated that a specific type of khat leads to an increase in the level of ALT^25^. This discrepancy may be due to the type of khat and the duration of khat chewing.

In the current study, there was no statistically significant difference in the levels of ALP among khat chewers compared to non-khat chewers. Similar results were also reported in Yemen^24^ and another study conducted in Yemen^20^ and Jazan region of Saudi Arabia^18^. However, the current study’s finding of ALP contradicts the study done in Kenya^26^, which indicated statistically significantly higher ALP level among khat chewers compared to non-khat chewers. The variation may be due to sex or age differences.

The TP levels had no statistically significant difference between khat chewers and non-khat chewers in the current study. This study’s finding of the TP levels was in line with the study conducted in Yemen^20^ and another study conducted in Yemen^24^. This may be due to the serum total protein level used to assess liver synthetic capacity, which will decrease in late-stage liver disease. But results from Kenya^26^ were contradictory to the present result. The difference may be attributed to the geographical location of the study participants or due to differences in nutritional status.

The current study found no statistically significant difference in the levels of total bilirubin (TBI) and direct bilirubin (DBI) among khat chewers and non-khat chewers. This was supported by the study conducted in Yemen^24^ and another study conducted in Yemen^20^. Contrary to our study’s finding of direct bilirubin level, the study conducted in Kenya showed significantly lower DBI among khat chewers when compared to non-khat chewers^26^.The variation may be due to doses (bundle used), duration of khat consumption or sample size.

Our study showed a higher FBG level among khat chewers when compared to non-khat chewers. The findings of this study were supported by the surveillance study conducted in nine regions and two administrative cities of Ethiopia^27^ and Egypt^28^. The higher FBG level among khat chewers compared to non-khat chewers can be explained by the pharmacological action of khat, which involves elevated sympathomimetic activity through elevated catecholamines, which inhibit insulin action and elevate glucagon secretion. The stimulation of skeletal muscle and liver glycogenolysis results in an elevated blood glucose level^29,30^. However, a study conducted in Ethiopia^31^ and Yemen^32^ demonstrated a significant decrease in fasting blood glucose level among khat chewers when compared to non-khat chewers, which contradicts the finding of the fasting blood glucose level of the present study. A study conducted in Yemen^33^ and another study from Yemen^34^ also reported a non-significant difference in FBG level among khat chewers compared to non-khat chewers. This variation may be due to differences in the type of khat and variations in lifestyle^35^.

With respect to the association between khat chewing patterns and liver function tests and fasting blood glucose level, this study demonstrated that both transaminase enzymes and fasting blood glucose levels were significantly and positively correlated with the duration of khat chewing. This relationship suggests that prolonged khat use may contribute to progressive hepatocellular injury and the development of hyperglycemia^36,37^. A study conducted in Yemen also indicated that the duration of khat chewing had a significant effect on the level of ALT^24^. There was only a weak correlation between the duration, frequency , and bundle of the khat with AST level. The lack of stronger correlation might be because AST is a mitochondrial enzyme and as such would not be easily affected by exogenous factors like khat.

## Conclusion and recommendation

The current study indicated that transaminase enzymes (AST and ALT) and fasting blood glucose (FBG) were higher among khat chewers compared to non-khat chewers. Duration, frequency and bundle of khat also had a positive correlation with transaminase enzymes (AST and ALT) and fasting blood glucose (FBG). We recommend a longitudinal study to be done among chronic khat chewers to identify the causal relationship of khat with liver function tests and fasting blood glucose levels. We recommend that future studies incorporate HbA1c and GGT to better evaluate the long-term impact of khat chewing on glycemic control and liver. As drug abuse is a great public health concern, health education should be promoted to the community.

### Limitation of the study

The limitations of the current study primarily involve the cross-sectional design that prevents establishing a cause-and-effect relationship, while the exclusion of chronic khat chewers with pre-existing conditions may have introduced a negative bias. HA1c and GGT were not done due to resource limitation. Despite the noted limitations, this study offers valuable insights given the lack of available data in Ethiopia.

## Data Availability

The reasonable requests dataset for this article can be accessed from the corresponding author.

## Abbreviations

ALP: Alkaline phosphatase
ALT: Alanine aminotransferase
AST: Aspartate aminotransferase
BMI: Body mass index
DBI: Direct bilirubin
DBP: Diastolic blood pressure
FBG: Fasting blood glucose
KC: Khat chewers
NKC: Non khat chewers
SBP: Systolic blood pressure
TBI: Total bilirubin
TP: Total protein
WC: Waist circumference
